# ﻿A new species of genus *Acrossus* Mulsant, 1842 (Scarabaeidae, Aphodiinae, Aphodiini) from South Korea

**DOI:** 10.3897/zookeys.1211.118456

**Published:** 2024-09-04

**Authors:** Changseob Lim, Łukasz Minkina

**Affiliations:** 1 Ojeong Resilience Institute, Korea University, Seoul, Republic of Korea; 2 Korean Entomological Institute, Korea University, Seoul, Republic of Korea; 3 os. Polana Szaflarska 4/39, 34-400 Nowy Targ, Poland

**Keywords:** Coleoptera, DNA barcode, Korean fauna, small dung beetles, taxonomy

## Abstract

A new species of the genus *Acrossus* Mulsant, 1842, *Acrossusbaei***sp. nov.** from South Korea, is described and illustrated on the basis of morphology and mitochondrial *COI* sequences. The species was compared with four related species; *Acrossusatratus* (Waterhouse, 1875), *A.humerospinosus* (Petrovitz, 1958), *A.luridus* (Fabricius, 1775), and *A.superatratus* (Nomura & Nakane, 1951). The taxonomic status and diagnostic characters of the new species are discussed. A key to species of the genus *Acrossus* in the Korean Peninsula is given.

## ﻿Introduction

*Acrossus* Mulsant, 1842 is a species-rich genus with 43 species known to date, one of which has two subspecies. Most of *Acrossus* species were originally described in the genus *Aphodius* Hellwig, 1798, where they have sometimes been placed in the subgenus Acrossus. Dellacasa et al. (2016) elevated the rank of *Acrossus* from subgenus to genus. The genus includes medium-sized to large species found mainly in the Palearctic and Oriental regions. One species is known from North America, and one from the Afrotropical region (Dellacasa et al. 2016). Most species of this genus have a very distinctive feature: an anteriorly rounded or truncate clypeus, and, for this reason, in the past many species now belonging to other genera (such as *Paracrossidius* Balthasar, 1932 or *Odontacrossus* Dellacasa G., Král, Dellacasa M. & Bordat, 2014) were erroneously placed here in *Acrossus*. Some species (e.g. *A.devabhumi* (Mittal, 1993)) still have a questionable position within the genus. In the last 20 years, only two species have been newly described in the genus: *A.byki* Minkina, 2018 and *A.jeloneki* Minkina, 2018. The genus *Acrossus* still needs research due to the unsatisfactory level of knowledge of its species diversity.

The first author, during his study of the Aphodiinae from South Korea, found several specimens of *Acrossus* he could not identify with available literature and which, after careful examination, proved to be an undescribed species. Here, we describe it as *Acrossusbaei* sp. nov. based on a morphological comparison with the most similar species (*A.atratus* (Waterhouse, 1875), *A.humerospinosus* (Petrovitz, 1958), *A.luridus* (Fabricius, 1775), and *A.superatratus* (Nomura & Nakane, 1951)) and a phylogenetic analysis of *COI* gene sequences. A key to the genus *Acrossus* in the Korean Peninsula is also provided.

## ﻿Material and methods

### ﻿Specimen sampling and examination

Adult dung beetles were collected using bait-traps with various animal feces or a flight interception trap (FIT). The specimens were observed with a Nikon SMZ-U stereomicroscope. The photos were taken by a Canon EOS 5D Mark III camera equipped with a Canon MP-E 65 mm macro lens (Tokyo, Japan). Photographs were combined in Helicon Focus 7 and Adobe Photoshop Elements 2018 software. For morphological terms used in the description of species, we follow Dellacasa et al. (2001) and Dellacasa et al. (2010). The type series of the new species are indicated by a red, printed label bearing the status of the specimen, sex, name, authorship, and the year and month of the designation.

The type series and examined specimens are a part of following collections:

**KUEM**Korea University Entomological Museum (South Korea)

**NIBR**National Institute of Biological Resources (South Korea)

**SEHU**Hokkaido University Museum (Japan)

**ABCP** Axel Bellmann, private collection (Germany)

**ISEA** Łukasz Minkina and Zdzisława Stebnicka collection deposited in Institute of Systematics and Evolution of Animals Polish Academy of Sciences in Kraków (Poland)

### ﻿Phylogenetic analysis

Total genomic DNA was extracted from the leg tissues of beetles using DNeasy Blood & Tissue Kit (Qiagen, Hilden, Germany) according to the manufacturer’s instruction. *COI* sequences were amplified using a primer set C1-J-2183 (5′ CAA CAT TTA TTT TGA TTT TTT GG 3′) and TL2-N-3014 (5′ TCC AAT GCA CTA ATC TGC CAT ATT A 3′) (Simon et al. 1994) with AccuPower® PCR PreMix (Bioneer, Daejeon, South Korea). A new primer set Acr-L1 (5′ GCC GGG ATA CCT CGA CGA TAC T 3′) and Acr-R1 (5′ TGC TCT GCA GGA GGC ATT TGT 3′) was specifically designed to amplify sequences for old museum specimens. The polymerase chain reactions (PCR) were performed following condition: an initial denaturation for 3 min at 94 °C; followed by 36 cycles of denaturation for 30 sec at 94 °C, annealing for 30 sec at 48–50 °C and extension for 90 sec at 72 °C; and a final extension for 5 min at 72 °C. The quality of PCR amplification was verified by running the PCR products on 1.5% agarose gel electrophoresis. The verified PCR products were purified using Exonuclease I and Shrimp Alkaline Phosphatase (New England BioLabs, Ipswich, MA, USA) and then sequenced by Macrogen INC (South Korea) on an ABI Prism® 3130 Genetic Analyzer (Applied Biosystems, Foster City, CA, USA) following a standard sequencing protocol. Sequences were aligned using the ClustalW algorithm in MEGA v. 10.2.6 (Kumar et al. 2018) and subsequently submitted to GenBank (accession numbers OR621067–OR621075, PP933827–PP933829). In total, 754 bp were obtained for the phylogenetic analysis, except for *A.atratus* and *A.humerospinosus*, which only had 220 bp available. The partial deletion method was adopted for these species in the subsequent analysis. This method allows us to retain the informative sites within the remaining 534 bp, thereby minimizing the impact of missing data on the accuracy and robustness of the phylogenetic analysis (Nei and Kumar 2000).

A total of 19 *COI* sequences were used for the phylogenetic analysis. These sequences included 12 newly obtained sequences of *A.baei* sp. nov. (seven sequences), *A.superatratus* (two sequences), *A.atratus* (two sequences) and *A.humerospinosus* (one sequence) as well as six GenBank sequences (AY132409, AY132509–AY132511, MH020527, MT872705) representing four *Acrossus* species (*A.depressus*, *A.luridus*, *A.carpetanus*, and *A.rufipes*). *Nimbusaffinis* (Panzer, 1823) (AY132590) was included as the outgroup, following the phylogenetic relationships proposed by Cabrero‐Sañudo and Zardoya (2004). The genetic divergence of the sequences was estimated as *p*-distance in MEGA v. 10.2.6. Maximum-likelihood (ML) and neighbor-joining (NJ) analyses were performed for the phylogenetic reconstruction. The GTR + I model was selected by the best evolutionary substitution model by jModelTest v. 2.1.7 (Darriba et al. 2012) based on Akaike information criterion (AICc). ML and NJ were performed using IQ-TREE webserver (Trifinopoulos et al. 2016) and MEGA, respectively, with 1000 bootstrap replicates.

## ﻿Taxonomy

### ﻿ Acrossus baei sp. nov.

Taxon classificationAnimaliaColeopteraScarabaeidae

4ABDE025-C68F-557B-A97E-50711C9B213E

https://zoobank.org/3171E85C-856D-4BE3-A5C1-B217E35ED539

Figs 1–3 , 17 , 22 , 23 , 32 , 37 , 42 , 47 , 52 , 57 Korean name: 산마루똥풍뎅이 (San-ma-ru-ddong-pung-deng-i)

#### Diagnosis.

The new species can be classified as *Acrossus* (following Dellacasa et al. 2001) due to: body moderately convex; head wide, eyes small, frontal suture not tuberculate; pronotum basally and laterally not serrulate, with sides always visible from above, basally and anteriorly not bordered, with posterior angles weakly obtuse-angled, with sides glabrous; scutellum small, triangular, flat, wider than two first intervals; elytra with ten distinct, impressed striae, part of them joined together before apex, humeral denticles small but distinct, intervals with distinct macrosetation; abdominal ventrites not fused each other; meso- and metatibiae apically fimbriate with spinules of unequal length .

The new species can be distinguished from all other known *Acrossus* species by the combination of the following features: moderately large body length (6.0–7.4 mm); body blackish, elytra rarely with orange spots before apex (last colour form is quite unique in the genus); head large, clypeus weakly sinuate anteriorly (only *A.humerospinosus* (Petrovitz, 1958) have that feature); pronotum wider than base of elytra; punctation of pronotum double, dense, coarser punctures with at about three times larger diameter than smaller ones (there is not to many species with so coarse and dense punctation of pronotum); humeral denticles small but distinct (this feature helps to distinguish it from somewhat similar species: *A.atratus* and *A.luridus*); whole elytra with distinct, long macrosetation (there is no other species with so long, distinct macrosetation on whole elytral surface), intervals with very dense and coarse punctation (unique feature); elytra before apex with distinct microreticulation, matt; male’s apical spur of protibiae distinctly inwardly hooked (however hook is better visible from bottom side, and in old specimens it can be wiped out, then apex of apical spur looks for widely rounded, but still inwardly curved); with two or three small teeth between first and second teeth of protibiae and three to five small teeth between second and third teeth of protibiae (this feature help to distinguish it from somewhat similar species: *A.atratus* and *A.luridus*). Aedeagus at apex with small membranous process visible only in lateral view. For more details and links to the photographs see Table 1 and Discussion.

**Figures 1–3.**
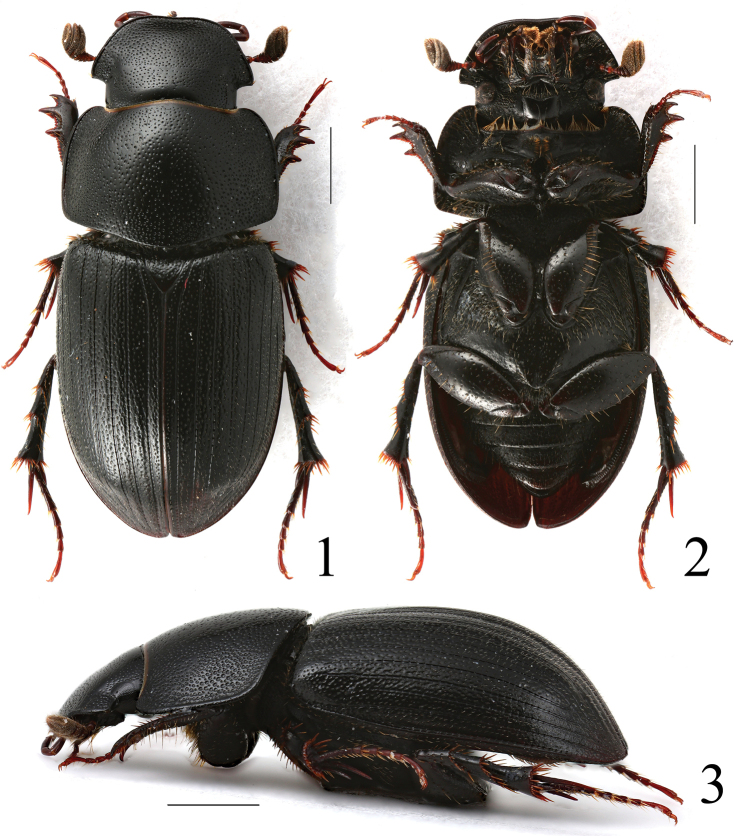
*Acrossusbaei* sp. nov., ♂, holotype **1** dorsal view **2** ventral view **3** lateral view. Scale bars: 1.0 mm.

**Table 1.** Differential characteristics of *Acrossus* species potentially confused with *A.baei* sp. nov.

Feature / species	*Acrossusbaei* sp. nov.	*Acrossusatratus* (Waterhouse, 1875)	*Acrossushumerospinosus* (Petrovitz, 1958)	*Acrossusluridus* (Fabricius, 1775)	*Acrossussuperatratus* (Nomura & Nakane, 1951)
Colour of elytra	Blackish, sometimes with orange-brownish spots before apex (Figs 1, 57)	Blackish (Fig. 5)	Blackish, very rarely basal half of elytra yellowish-brown (Fig. 8)	Very variable: from blackish with a lot of yellowish strips to totally blackish (Fig. 11)	Blackish (Fig. 14)
Convexity of body	Relatively least convex (Fig. 3)	Distinctly convex (Fig. 7)	Distinctly convex (Fig. 10)	Relatively less distinctly convex (Fig. 13)	Distinctly convex (Fig. 16)
Anterior part of clypeus	Weakly sinuate (Fig. 17)	Truncate (Fig. 18)	Weakly sinuate (Fig. 19)	Truncate (Fig. 20)	Truncate, sometimes weakly sinuate (Fig. 21)
Punctation of clypeus	Very dense, punctation double (Fig. 17)	Very dense, punctation double (Fig. 18)	Very dense, punctation simple (Fig. 19)	Dense, punctation simple (Fig. 20)	Very dense, punctation double (Fig. 21)
Apical spur of protibiae in male	Distinctly inwardly hooked before apex; when visible from above situated on inner side of protibiae, when visible anteriorly seems to be rounded at apex; in old specimens its visible only as elongate, weakly inwardly curved spur with widely rounded apex (Fig. 37)	Distinctly inwardly hooked before apex; when visible from above situated on inner side of protibiae, when visible anteriorly seems to be rounded at apex; in old specimens its visible only as elongate, weakly inwardly curved spur with widely rounded apex (Fig. 38)	When visible from above situated on upper side of protibiae and visible as acute at apex; when visible anteriorly distinctly acute at apex; in old specimens its visible as elongate, outwardly curved spur, still acute at apex (Fig. 39)	Distinctly downwardly directed, weakly inwardly hooked before apex; when visible from above situated on inner side of protibiae, when visible anteriorly seems to be rounded at apex; in old specimens its visible only as elongate, still distinctly inwardly curved spur with widely rounded apex (Fig. 40)	When visible from above situated on upper side of protibiae, and visible as acute at apex; when visible anteriorly distinctly acute at apex; in old specimens its visible as elongate, weakly outwardly curved spur, still acute at apex (Fig. 41)
Number of small teeth between first and second teeth and between second and third teeth	2–3 / 3–5 (Fig. 37)	1–2 / 1–2 (Fig. 38)	1–3 / 2–5 (Fig. 39)	0 / 0 (Fig. 40)	2–3 / 3–5 (Fig. 41)
Sides of pronotum	Widely rounded (Fig. 1)	Truncate (Fig. 5)	Widely rounded (Fig. 8)	Widely rounded (Fig. 11)	Widely rounded (Fig. 14)
Punctation of elytra	Very dense, distinctly coarse (Fig. 47)	Very dense, moderately coarse (Fig. 48)	Very dense, moderately coarse (Fig. 49)	Very dense, moderately coarse (Fig. 50)	Very dense, moderately coarse (Fig. 51)
Humeral denticles on elytra	Small but distinct (Fig. 1)	Absent (Fig. 5)	Large, distinct (Fig. 8)	Absent (Fig. 11)	Small but distinct (Fig. 14)
Macrosetation of elytra	Long macrosetae on whole surface of elytra except disc, where are slightly shorter (Figs 1, 42)	Long macrosetae on whole surface of elytra except disc, where usually are distinctly shorter (Figs 5, 43)	Long macrosetae only on sides and before apex; very short macrosetae on whole surface of elytra (Figs 8, 44)	Long macrosetae only on sides and before apex; microsetae (visible at 200× magnification nearly on whole surface of elytra) (Figs 11, 45)	Long macrosetae on whole surface of elytra except disc, where usually are distinctly shorter (Figs 14, 46)
Apex of elytra	With relatively low preapical declivity (Fig. 3), with very distinct microreticulation (see left elytron), matt (compare with right elytron) (Fig. 42)	With relatively high preapical declivity (Fig. 7), with relatively weak microreticulation (see left elytron), shiny (compare with right elytron) (Fig. 43)	With relatively high preapical declivity (Fig. 10), without microreticulation (see left elytron), shiny (compare with right elytron) (Fig. 44)	With relatively low preapical declivity (Fig. 13), with distinct microreticulation (see left elytron), matt, (compare with right elytron) (Fig. 45)	With moderately high preapical declivity (Fig. 16), with weak microreticulation (see left elytron), matt (compare with right elytron) (Fig. 46)
Shape of metatibial claws	Moderately large, fourth metatarsomer more than two times long as their claw (Fig. 52)	Small, fourth metatarsomer nearly three times long as their claw (Fig. 53)	Moderately large, fourth metatarsomer more than two times long as their claw (Fig. 54)	Large, fourth metatarsomer less than two times longer as their claw (Fig. 55)	Small, fourth metatarsomer nearly three times long as their claw (Fig. 56)
Shape of epitorma with amount of angustofenestrae (celtes) on top	Epitorma elongate, thin, fully developed; 3 angustfenestrae on top (Fig. 32)	Epitorma elongate, relatively wide, shortened to 3/4 of length; 1 angustfenestra near top (Fig. 33)	Epitorma elongate, relatively wide, shortened to 7/8 of length; 3 angustofenestrae near top (Fig. 34)	Lack of epitorma; 1 angustofenestra at apex of row with angustofenestrae (Fig. 35)	Epitorma elongate, thin, fully developed; 2 angustfenestrae on top (Fig. 36)
Shape of aedeagus	At apex, on sides with very weak membranous process visible only in lateral view (Figs 22, 23)	At apex, on sides with very distinct, weakly sclerotized membranous process visible from above (Figs 24, 25)	At apex, on sides with distinct membranous process visible from above (Figs 26, 27)	At apex without any membranous process (Figs 28, 29)	At apex, on sides with distinct membranous process visible from above (Figs 30, 31)
Distribution	South Korea	Japan	China (Sichuan)	Europe, North Africa (Morocco), Kazakhstan, Kyrgyzstan, Russia (West Siberia), China (Xinjiang)	Russia (East Siberia and Far East), Japan, North Korea, South Korea

#### Type locality.

South Korea, Gangwon-do, Pyeongchang-gun, Jinbu-myeon, Mountain Odaesan.

#### Type materials.

***Holotype***: South Korea • ♂; Gangwon-do, Pyeongchang-gun, Jinbu-myeon, Mt. Odaesan; 37°47.23'N, 128°33.91'E; alt. 1000 m; 18 Apr.–01 May 2020; C. Lim leg.; KUEM.

***Paratypes*** (10 spm.): South Korea • 5 spm.; same data as holotype; 2 ♂, ♀ ISEA; ♂ABCP; ♂ NIBR • 2 ♂, ♀; Gangwon-do, Hongcheon-gun, Nae-myeon, Mt. Gyebangsan; 37°44.78'N, 128°25.68'E; alt. 830 m; 30 Apr. 2020; C. Lim leg.; GenBank: OR621067, OR621069–OR621070; CSL-21-0013–CSL-21-0015; KUEM • ♀; Gangwon-do, Hongcheon-gun, Nae-myeon, Mt. Gyebangsan; 37°44.78'N, 128°25.68'E; alt. 830 m; 30 Apr. 2020; C. Lim leg.; KUEM • ♀; Gyeongsangbuk-do, Youngju-si, Punggi-eup, Mt. Sobaeksan; 36°56.23'N, 128°27.6'E; alt. 856 m; 05 May 2019; C. Lim leg.; KUEM.

#### Additional materials.

South Korea • ♂, 2 ♀; Jeju-do, Seogwipo-si, Jungmun-dong, Youngsil trail; 33°20.2'N, 126°28.1'E; 17–27 Mar. 2021; C. Lim, J. Kim, J.M. Hwang, D. Lee legs.; GenBank: OR621071–OR621073; CSL-21-0083–CSL-21-0085; KUEM • ♀; Jeju-si, Nohyung-dong; 33°25.19.1'N, 126°29.31.6'E; 06 Jun. 2019; C. Lim leg.; GenBank: OR621068; CSL-21-0071; KUEM.

#### Description.

***Dorsum*** (Fig. 1). Moderately large species, relatively small as a member of the genus, body length of the holotype 6.7 mm; elongate-oval, shiny, blackish; antennae, tarsomeres, and mouth parts reddish brown.

***Head*** (Fig. 17) large, distinctly widely trapezoidal, convex, shiny, without microreticulation. Clypeus distinctly bordered, weakly sinuate anteriorly, widely rounded laterally, not notched before genae, clypeal border without macrosetae. Genae acute-angled, very distinctly exceeding eyes, with few relatively short, thin macrosetae in basal part. Frontal suture not marked, but visible as surface without punctation, without gibbosities, epistoma without gibbosity. Punctation double, but both kinds of punctation not so clearly distinguishable due diameter of larger punctures being only two times larger than smaller ones; both kinds of punctation quite regularly, densely distributed; punctures somewhat variable in size; genae with much denser punctation.

***Epipharynx*** (Fig. 32) transverse, with sides distinctly rounded, anterior margin of concavely arcuate, corypha not developed, zygum very narrow, with three long, thick angusto-fenestrae at apex and three additional ones arranged as row. Acanthopariae with dense, long, thin chaetae; acropariae with dense, short, thinner chaetae than on acanthopariae; chaetopariae with dense belt of quite thin, quite short chaetae; adelochaetae absent; prophobae with dense, short, thin macrosetae; chaetopediae absent. Epitorma reduced to a small, narrow triangle. Tormae relatively thin, long.

***Pronotum*** transverse, somewhat wider than base of elytra, widest near base, moderately convex, shiny, without microreticulation, with double punctation; smaller punctures fine, with diameter about three times smaller than large punctures, quite regularly distributed, dense; larger punctures coarse, dense, not regularly distributed, much denser near base and on sides. Pronotum anteriorly and basally not bordered, distinctly bordered on sides. Borders without macrosetae. Anterior angles widely rounded; posterior angles weakly obtuse-angled, base before posterior angles truncate.

***Scutellum*** small, triangular, with dense, irregularly sized punctation, moderately shiny, with distinct microreticulation.

***Elytra*** (Fig. 47) elongate-oval, convex, widely rounded, weakly shiny, with weak microreticulation on disc, becoming much more distinct on sides and apex, with small but distinct humeral denticles; with ten striae and ten intervals. Striae distinctly, quite sparsely punctate with moderately large punctures; punctures weakly but clearly indenting margins of intervals. First and tenth, third and fourth, fifth and sixth striae joined together before apex, sixth to eighth striae shortened before base, eighth distinctly; ninth and tenth striae joined before base. Intervals weakly shiny, very weakly convex, with irregularly distributed simple moderately coarse punctation, this irregular in size. Almost all punctures (on disc somewhat less frequently) bearing short, thin macrosetae.

***Pygidium*** with similar sculpture as on abdominal ventrites.

***Legs*.** Femora shiny, without microreticulation, quite finely and densely punctate, with punctures bearing short macrosetae. Profemora basally and apically with a belt of punctures bearing very long macrosetae; mesofemora basally with a belt of punctures bearing very long macrosetae, metafemora with much sparser than on mesofemora row of punctures bearing long macrosetae apically. Protibiae (Fig. 37) distinctly tridentate laterally, proximally serrulate; additional few (2–3) small teeth between first and second teeth, and additional few (3–5) small teeth between second and third teeth; dorsal side smooth, shiny, with a few fine punctures bearing short macrosetae; apical spur long, moderately broad, straightforward, distinctly downwardly and inwardly hooked before apex. Meso- and metatibiae with two distinct transverse carinae, fimbriate apically with row of long spinules of unequal length. Metatibiae superior apical spur very slightly longer than basimetatarsomere, latter distinctly longer than 3½ of next metatarsomeres combined. Claws (Fig. 52) moderately long, moderately thick, moderately arcuate.

***Macropterous*.** Venter (Fig. 2). Meso-metaventral plate shiny, very slightly concave, with indistinct, quite shallow longitudinal concavity in the middle and weak longitudinal line in the middle; surface with variable in size, shallow, irregularly spaced, not so dense punctation, bearing short, thin macrosetation. Abdominal ventrites matte, with very distinct microreticulation, with quite dense, fine punctures bearing moderately long, thin macrosetae; additionally last abdominal ventrite, in the middle with row of punctures bearing very long macrosetae.

***Aedeagus*** (Figs 22, 23) with parameres slightly shorter than phallobase. Parameres weakly but regularly downwardly bent; at apex, on sides with very weak membranous process, additionally with few very thin and very short macrosetae, which are directed inwardly and not visible due to the time when we try to separate parameres.

#### Etymology.

The species is named in honor of Dr Yeon Jae Bae who has contributed to the conservation of dung beetles in South Korea.

#### Sexual dimorphism.

Males with apical spur of anterior tibiae distinctly downwardly and inwardly hooked before apex, meso-metaventral very slightly concave. Females with apical spur acute at apex, meso-metaventral plate very weakly convex.

#### Variability.

Size from 6.0 to 7.5 mm. Elytra usually blackish, sometimes with short, orange-brownish stripes before apex (Fig. 57). Punctation of head and pronotum weakly variable. Connection between elytra striae of elytra somewhat variable.

#### Remark.

**Figure 4.**
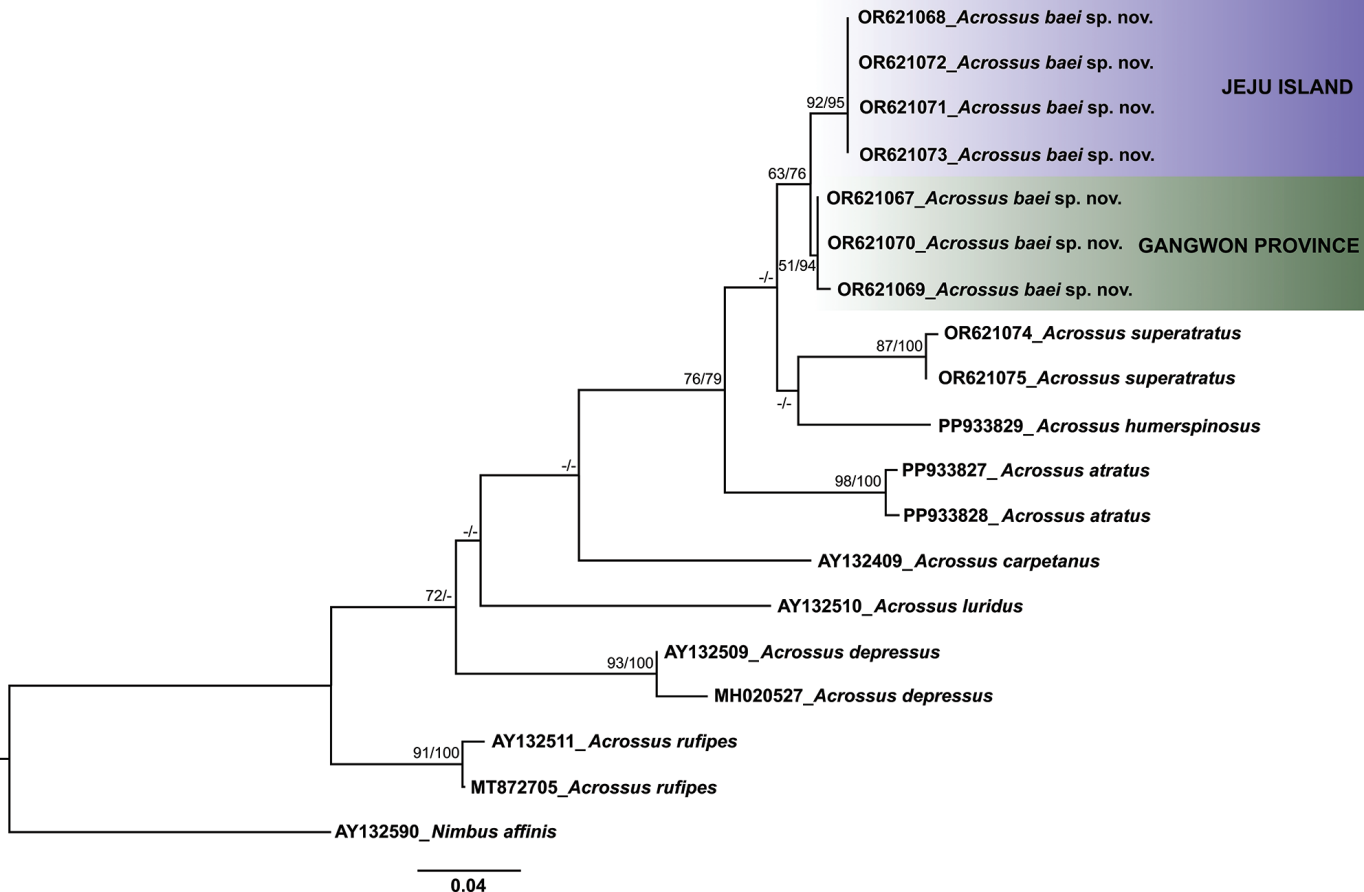
Phylogenetic tree based on 19 mitochondrial *COI* gene sequences of the eight *Acrossus* species and *Nimbusaffinis* (outgroup). Branch values indicate bootstrap support in maximum likelihood (ML) and neighbor joining (NJ), respectively. Tree topology and branch lengths reflect the results of ML analysis. The tree is drawn to scale, with branch lengths (evolutionary distance) measured in the number of substitutions per site. Dashes (–) indicate support values of less than 50 or incongruent between ML and NJ.

We have decided that part of the material of *A.baei* sp. nov. should be excluded from the type series. Based on a shortage of comparative material, we cannot determine the exact range of inter-individual variability of the population from Jeju Island. Therefore, in our opinion, it is better to identify type material from only one specific location (i.e. mainland South Korea)(Fig. 4).

### ﻿ Acrossus atratus

Taxon classificationAnimaliaColeopteraScarabaeidae

(Waterhouse, 1875)

8F946B71-A9B5-5778-A94F-16954EEB51F0

Figs 5–7 , 18 , 24 , 25 , 33 , 38 , 43 , 48 , 53

#### Materials.

Japan • ♂ (photographed); Saitama-ken, Asaka-shi, Adachi; 21 Apr. 1971; S. Nagao leg.; SEHU • 2 spm.; same data as photographed specimen; SEHU • 2 spm.; same data as photographed specimen; GenBank: PP933827–PP933828; CSL-21-0439–CSL-21-0440; SEHU • ♀; Kumamoto-ken, Aso-gun, Aso-shi, Mt. Oujou-dake; 26 Apr. 1999; S. Kawai leg.; ISEA.

### ﻿ Acrossus humerospinosus

Taxon classificationAnimaliaColeopteraScarabaeidae

(Petrovitz, 1958)

A23CB05A-12D2-54BC-9110-D14516DA201E

Figs 8–10 , 19 , 26 , 27 , 34 , 39 , 44 , 49 , 54

#### Materials.

China • ♂ (photographed); C Sichuan, Mt. Jinding; alt. 1500 m; 20 Jun. 2012; V. Patrikeev leg.; ISEA • 1 spm.; Yunnan, Baihanchwag, 50 km NW Lijiang; alt. 2400 m; 05 Jun. 2006; Vladimir Major leg.; ISEA • 1 spm.; Yunnan, 25 km S Zhonghian; alt. 3200 m; 14 Jun. 2006; Vladimir Major leg.; ISEA • 1 spm.; Sichuan, rd. Danba to Bomei, 35 km W Danba; alt. 2500–2700 m.; Jun.–Jul. 2007; Puchner leg.; ISEA • 1 spm.; C Sichuan, Maoxian env. Jinding Mt.; alt. 1600 m; 20 Jun. 2012; V. Patrikeev leg.; ISEA • 2 spm.; C Sichuan, Maoxian env. Jinding Mt.; alt. 1500 m; 20 Jun. 2012; V. Patrikeev leg.; ISEA • 2 spm.; NW Yunnan, Lijang pref., S. Yulongxue Shan Mts.; alt. 3200 m.; 16 Jun. 2016; V. Patrikeev leg.; ISEA; • 1 spm.; W. Sichuan, Mt. Yadling, W of Yading vill.; alt. 3850–4650 m; 5–11 May 2012; D. Kral leg.; GenBank: PP933829; CSL-21-0459; KUEM.

**Figures 5–7.**
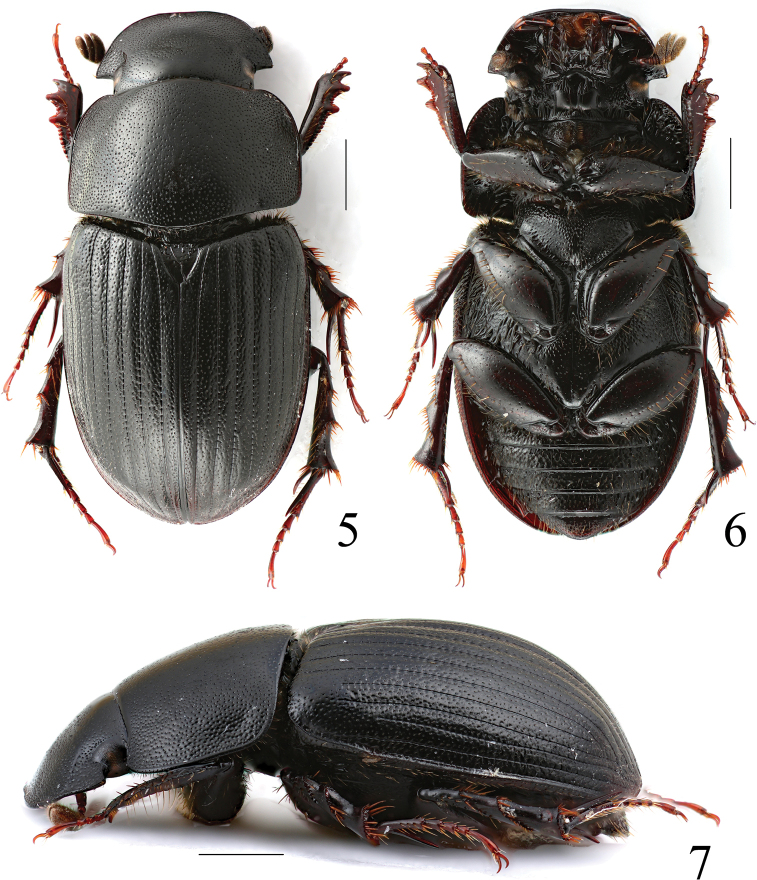
*Acrossusatratus* (Waterhouse, 1875), ♂ **5** dorsal view **6** ventral view **7** lateral view. Scale bars: 1.0 mm.

### ﻿ Acrossus luridus

Taxon classificationAnimaliaColeopteraScarabaeidae

(Fabricius, 1775)

F3CC35F2-B4F8-5486-946B-3CDB513EAD60

Figs 11–13 , 20 , 28 , 29 , 35 , 40 , 45 , 50 , 55

#### Materials.

Hungary • ♂ (photographed); Csernely; 22 Apr. 2012; Ł. Minkina leg.; ISEA • 4 spm.; same data as photographed specimen; ISEA. Ukraine • 1 spm.; płw. Tarchankut; 45°25'N, 32°32'E; 03 May 2008; C. Nowak leg.; ISEA. Bulgaria • 2 spm.; Yasna Polyana; 08 May 2013; Ł. Minkina leg.; ISEA. Iran • 1 spm.; Aarbaigan E, Sagri, 15 km W Nir; alt. 1750 m; 17 May 2002; P. Rapuzzi leg.; ISEA. Georgia • 2 spm.; Kartli Gomi; 41.905334°N, 44.380755°E, alt. 570–790 m; 5–21 May 2019; J. Klasinski leg.; ISEA. Turkey • 2 spm.; Antalia town, near Saklikent village; alt. 200 m; 1–3 May 2019; V. Patrikeev leg.; ISEA.

**Figures 8–10.**
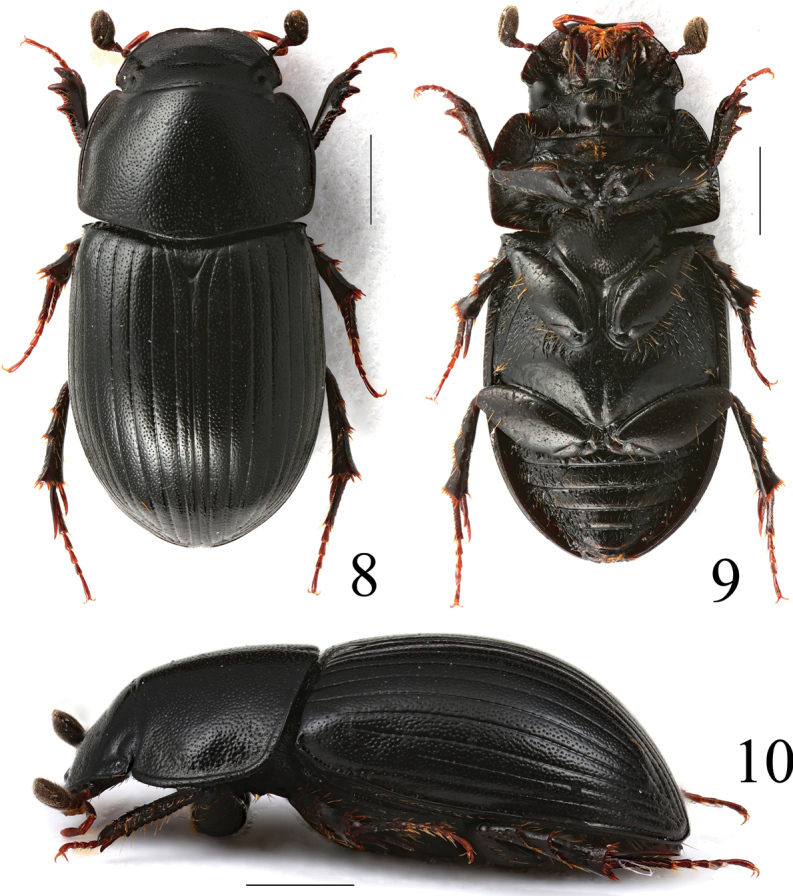
*Acrossushumerospinosus* (Petrovitz, 1958), ♂ **8** dorsal view **9** ventral view **10** lateral view. Scale bars: 1.0 mm.

**Figures 11–13.**
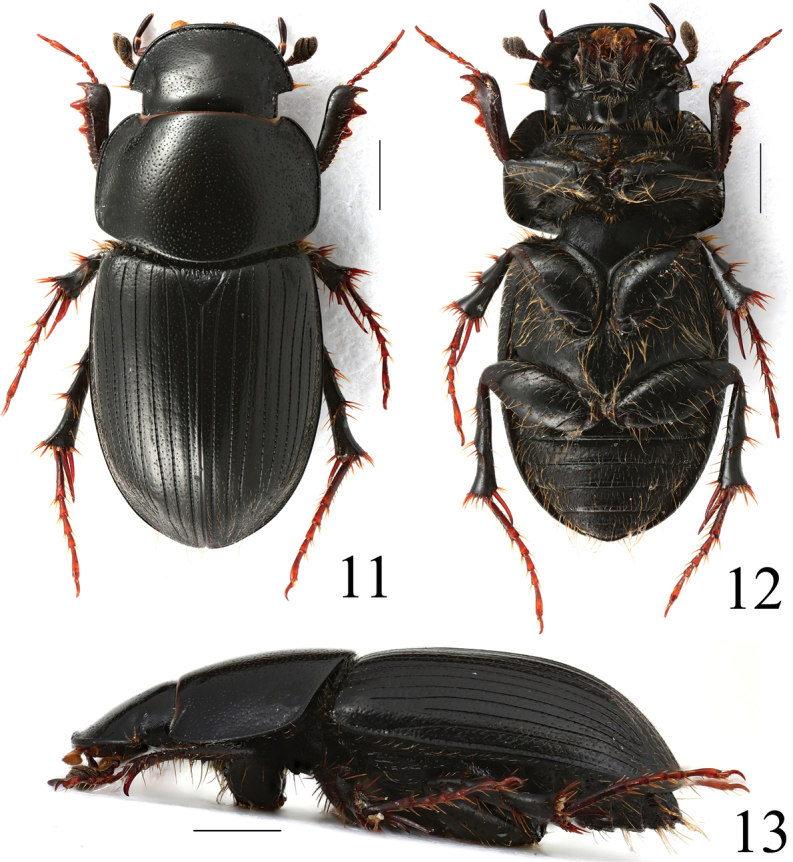
*Acrossusluridus* (Fabricius, 1775), ♂ **11** dorsal view **12** ventral view **13** lateral view. Scale bars: 1.0 mm.

### ﻿ Acrossus superatratus

Taxon classificationAnimaliaColeopteraScarabaeidae

(Nomura & Nakane, 1951)

CC02523C-E13E-5401-8208-16746620D2A0

Figs 14–16 , 21 , 30 , 31 , 36 , 41 , 46 , 51 , 56

#### Type materials.

***Holotype***: Japan • ♂; Honshu, Ise, Buhei-toge; 01 Jun. 1947; S. Osawa leg.; SEHU. ***Paratypes***: 2 spm.; same data as holotype; SEHU.

#### Additional materials.

Russia • ♂ (photographed); Far East, Primorskiy reg., Murav’ev-Amurskiy pen., Artem town env., Ozernyi kluch riv.; 15 May–10 Jun. 2005; A. Plutenko leg.; ISEA. North Korea • 13 spm.; Hamgjŏng-punkto prov., Kvanmo-bong (Mt., 60) at human excrements; 23 May 1974; Z. Stebnicka leg.; ISEA • 3 spm.; Ryanggang-do, Samjiyon; alt. 1000 m; 26 Jun. 1988; O. Merkl, Gy. Szel legs.; NIBR. South Korea • 1 spm.; Jeju-do, Jeju-si, Aewol-eup; 17 May. 1990; M.T. Chûjô leg.; NIBR • ♂; Gangwon-do, Hongchun-gun, Nae-myeon; 11 Jul. 1990; J.I. Kim leg.; NIBR • ♂; Yeongju-si, Mt. Sobaeksan, Huibanggyegok val.; 36°56.14'N, 128°27.37'E; 05 May. 2019; C. Lim leg.; KUEM • ♂; Pyeongchang-gun, Jinbu-myeon, Dongsan-ri, Mt. Odaesan, 1–29 May. 2020; C. Lim leg.; GenBank: OR621074; CSL-21-0429; KUEM • ♀; Seogwipo-si, Namwon-eup; 33°19.45'N, 126°36.22'E; 10 Jun. 2021; D.G. Kim leg.; GenBank: OR621075; CSL-21-0431; KUEM. Japan • 1 spm.; Ueno-Mura, Jukkoku-tóge Pass; 26 May 2001; S. Kawai leg.; ISEA • ♂; Kibune Yamashiro; May. 1948; K. Tsukamoto leg.; SEHU.

**Figures 14–16.**
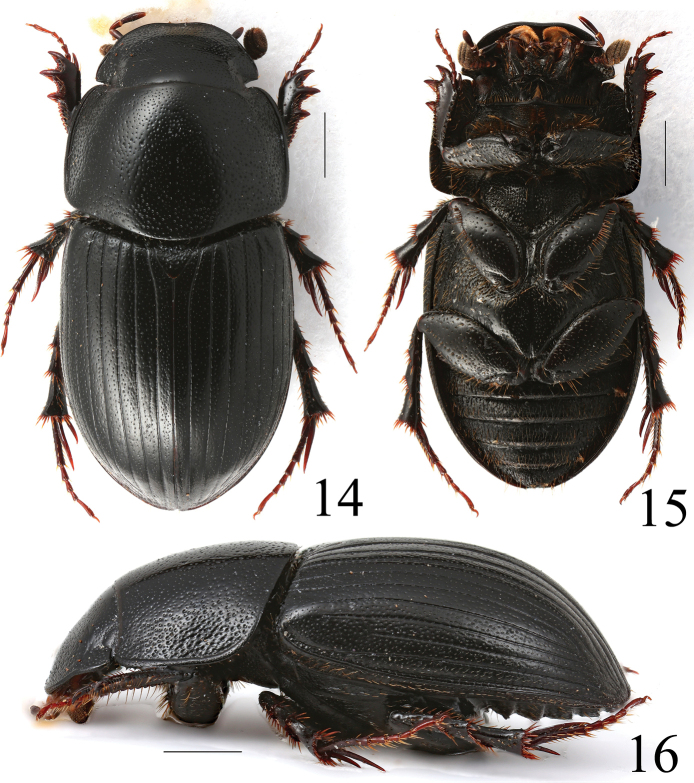
*Acrossussuperatratus* (Nomura & Nakane, 1951), ♂ **14** dorsal view **15** ventral view **16** lateral view. Scale bars: 1.0 mm.

##### ﻿Key to the species of *Acrossus* from the Korean Peninsula

**Table:** 

1	Elytra with distinct macrosetation (usually visible on sides and before apex at 50× magnification)	**2**
–	Elytra at most with indistinct macrosetation or glabrous (if visible, setation can be observed only on sides and before apex at 200× magnification)	**3**
2	Clypeus anteriorly weakly sinuate (Fig. 17). Apical spur of protibiae in males inwardly hooked before apex (Fig. 37). Elytra with clear macrosetation on whole surface (Figs 1, 3, 42, 47). Elytra before apex with very distinct microreticulation (Fig. 42). Punctation of body coarser	***Acrossusbaei* sp. nov.**
–	Clypeus anteriorly usually truncate, rarely weakly sinuate (Fig. 21). Apical spur of protibiae in males acute at apex (Fig. 41). Elytra with clear macrosetation on sides and before apex (Figs 14, 16, 46, 51). Elytra before apex with weak microreticulation (Fig. 46). Punctation of body finer	***Acrossussuperatratus* (Nomura & Nakane, 1951)**
3	Body brownish. Body length <6.5 mm or >10.0 mm. Elytra glabrous	**4**
–	Body blackish, frequently with lighter elytra. Body length 6.5–10.5 mm. Elytra with very short macrosetation before apex	**5**
4	Body oblong ovate, length less than 6.5 mm	***Acrossuskoreanensis* (Kim, 1986)**
–	Body elongate, length more than 10.0 mm	***Acrossusrufipes* (Linnaeus, 1758)**
5	Body length 7.0–10.0 mm. Body more deplanate, wider. Apical spur of protibiae in males more downwardly directed. Humeral denticles indistinct. Elytral intervals slightly more convex. Elytral punctation somewhat finer. Claws of hind legs more curved	***Acrossusbinaevulus* (Heyden, 1887)**
–	Body length 6.0–9.5 mm. Body less deplanate, narrower. Apical spur of protibiae in males less downwardly directed. Humeral denticles very small, but distinct. Elytral intervals slightly less convex. Elytral punctation somewhat coarser. Claws of hind legs less curved	***Acrossusdepressus* (Kugelann, 1792)**

##### ﻿Phylogenetic analysis and discussion

In the genus *Acrossus* Mulsant, 1842, the relations between species are still poorly known and the genus needs revision. According to our results, *A.baei* sp. nov. presents an adequately supported (63/76) monophyletic lineage (Fig. 4). Similarly, four other species—*A.rufipes*, *A.depressus*, *A.atratus*, and *A.superatratus*, each represented by at least two specimens—also present adequately supported monophyletic lineages. Among the five species we examined morphologically (excluding *A.luridus* for which only one sequence was used), four formed a monophyletic lineage (76/79). However, the phylogenetic relationships among the eight *Acrossus* species, including the relationship within the *A.baei* + *A.superatratus + A.humerospinosus + A.atratus* clade, were not well resolved in our tree. This could be attributed to the fact that our phylogenetic tree was reconstructed using only partial sequences from a single gene (*COI*). Further phylogenetic analysis, including multiple genes and a broader range of species, could offer better insights into the phylogenetic relationships within this genus.

**Figures 17–21.**
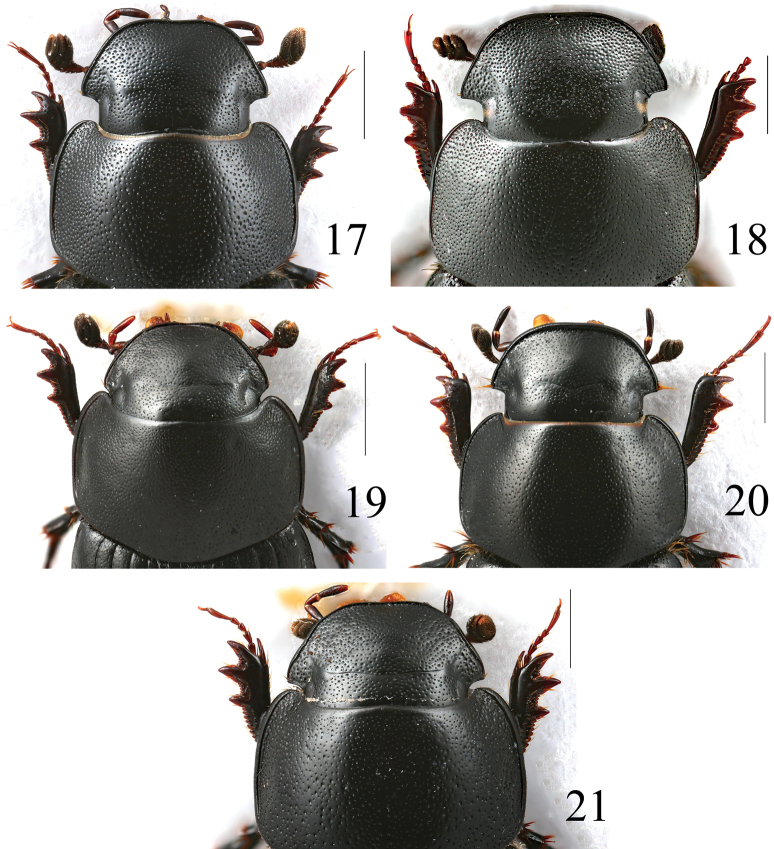
Heads of *Acrossus* species **17***A.baei* sp. nov., ♂, holotype **18***A.atratus* (Waterhouse, 1875), ♂ **19***A.humerospinosus* (Petrovitz, 1958), ♂ **20***A.luridus* (Fabricius, 1775), ♂ **21***A.superatratus* (Nomura & Nakane, 1951), ♂. Scale bars: 1.0 mm.

Intraspecific clades, consisting of individuals from two regions (Gangwon Province and Jeju Island) were observed in *A.baei* sp. nov. (genetic divergence: 1.42%; Table 2). The genetic divergence between *A.baei* sp. nov. and *A.superatratus*, based on individuals from the same regions, was 5.30%. This distance surpasses the typical species-level genetic divergence, indicating that they are distinct species. The other *Acrossus* species presented genetic differences ranging from 5.92% (*A.humerospinosus*) to 13.96% (*A.rufipes*) (Table 2).

**Table 2.** Estimates of genetic divergence (*p*-distance) between intraspecific clades of *A.baei* sp. nov., and between *A.baei* sp. nov. and seven other *Acrossus* species.

Group	Mean distance (%)	SE (%)
Between intraspecific clades of *A.baei* sp. nov. (Jeju vs Gangwon)	1.42	0.26
*A.superatratus*	5.30	1.39
*A.humerospinosus*	5.92	1.46
*A.atratus*	8.00	1.75
*A.luridus*	12.97	2.20
*A.depressus*	11.49	2.05
*A.rufipes*	13.96	2.24
*A.carpetanus*	11.06	2.06
Outgroup	15.73	2.45

*Acrossusbaei* sp. nov. is the third species known from South Korea (the two others are: *A.superatratus* (Nomura & Nakane, 1951) and *A.koreanensis* (Kim, 1986) (Stebnicka 1980; Kim 2012) and the sixth known from the Korean Peninsula (the three remaining are *A.binaevulus* (Heyden, 1887), *A.depressus* (Kugelann, 1792), and *A.rufipes* (Linnaeus, 1758)). Of all mentioned species, *A.baei* sp. nov. is most similar to *A.superatratus* in having distinct setation of the elytra. The other species have elytra with very short to indistinct setation (located mainly on sides of elytra or before their apices), or they are glabrous. To show the level of difference between them, we have proposed a key to the genus *Acrossus* from the Korean Peninsula.

**Figures 22–31.**
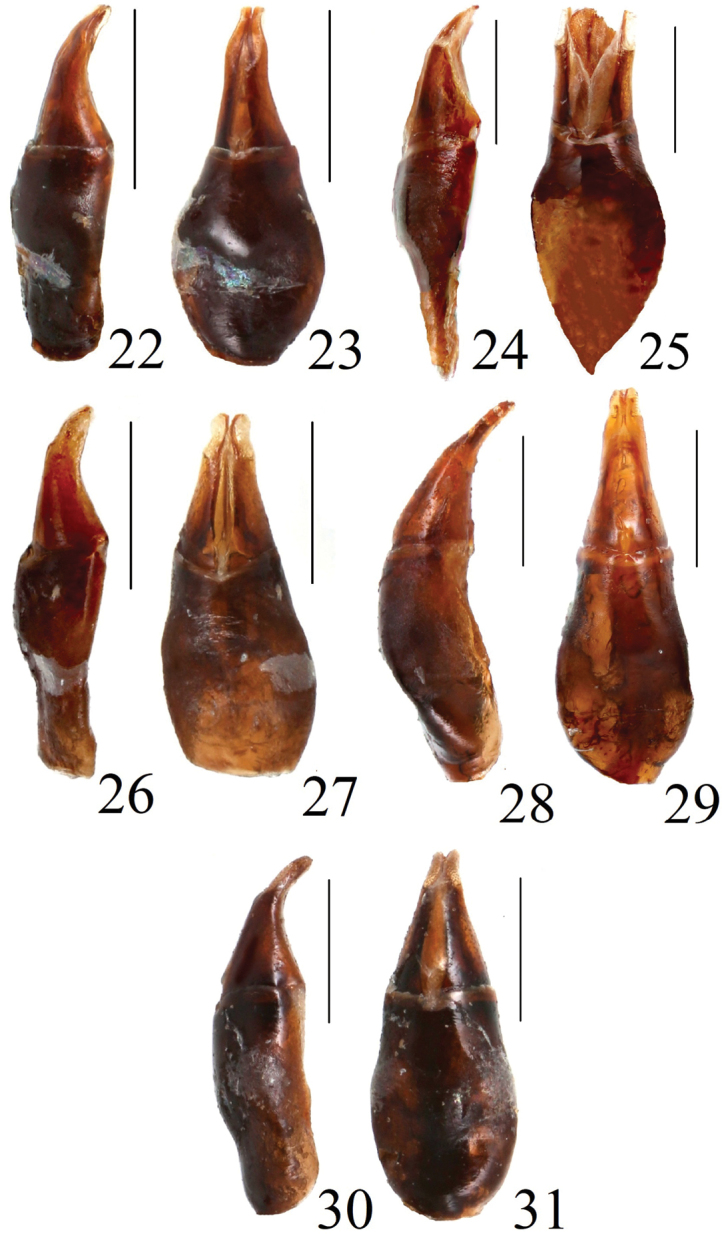
Aedeagi of *Acrossus* species **22***A.baei* sp. nov., holotype, lateral view **23***A.baei* sp. nov., holotype, dorsal view **24***A.atratus* (Waterhouse, 1875), ♂, lateral view **25***A.atratus* (Waterhouse, 1875), ♂, dorsal view **26***A.humerospinosus* (Petrovitz, 1958), lateral view **27***A.humerospinosus* (Petrovitz, 1958), dorsal view **28***A.luridus* (Fabricius, 1775), ♂, lateral view **29***A.luridus* (Fabricius, 1775), ♂, dorsal view **30***A.superatratus* (Nomura & Nakane, 1951), ♂, lateral view **31***A.superatratus* (Nomura & Nakane, 1951), ♂, dorsal view. Scale bars: 1.0 mm.

**Figures 32–36.**
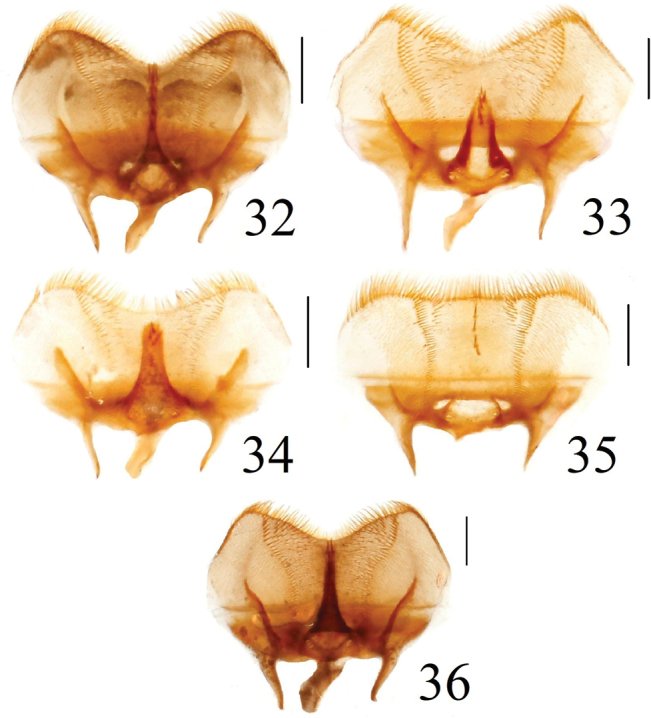
Epipharingi of *Acrossus* species **32***A.baei* sp. nov., ♂, holotype **33***A.atratus* (Waterhouse, 1875), ♂ **34***A.humerospinosus* (Petrovitz, 1958), ♂ **35***A.luridus* (Fabricius, 1775), ♂ **36***A.superatratus* (Nomura & Nakane, 1951), ♂. Scale bars: 0.5 mm.

**Figures 37–41.**
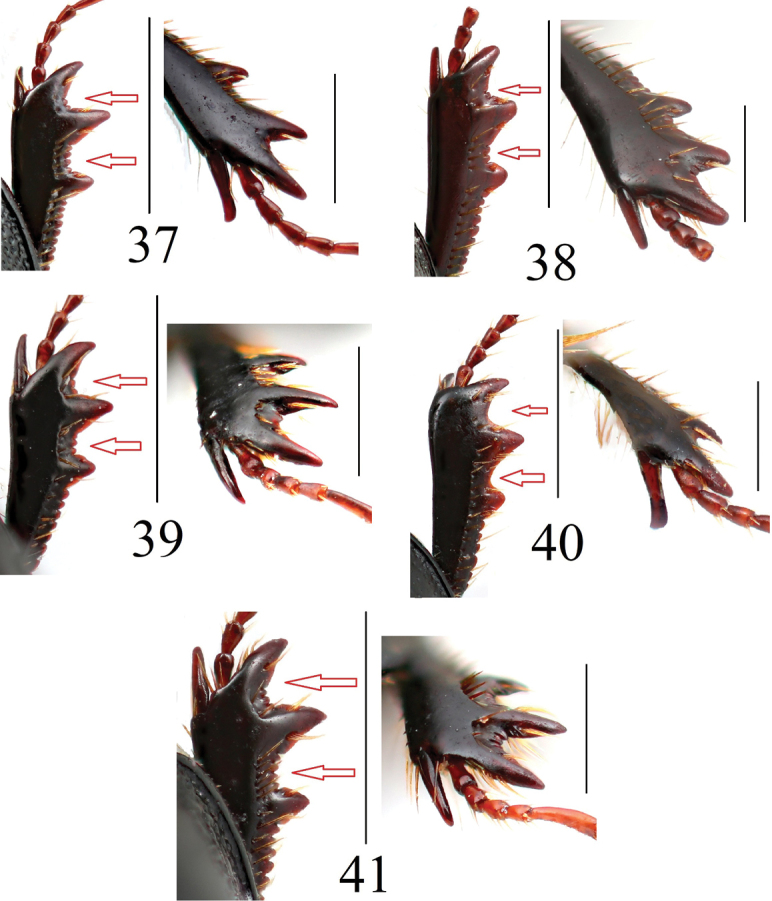
Protibiae and their apical spurs of *Acrossus* species **37***A.baei* sp. nov., ♂, holotype **38***A.atratus* (Waterhouse, 1875), ♂ **39***A.humerospinosus* (Petrovitz, 1958), ♂ **40***A.luridus* (Fabricius, 1775), ♂ **41***A.superatratus* (Nomura & Nakane, 1951), ♂. Scale bars: 1.0 mm.

**Figures 42–46.**
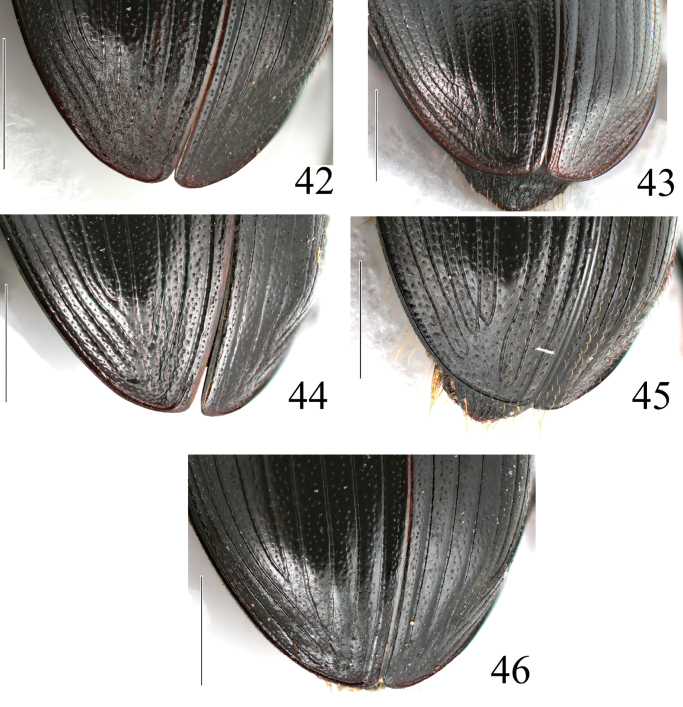
Apex of elytra of *Acrossus* species **42***A.baei* sp. nov., ♂, holotype **43***A.atratus* (Waterhouse, 1875), ♂ **44***A.humerospinosus* (Petrovitz, 1958), ♂ **45***A.luridus* (Fabricius, 1775), ♂ **46***A.superatratus* (Nomura & Nakane, 1951), ♂. Scale bars: 1.0 mm.

In having a blackish, longitudinally oval body, the apical protibial spur of the male flattened inwards or hooked at the apex, the elytra with distinct macrosetation, and the elytral intervals flat to weakly convex, *A.baei* sp. nov. is most similar to *A.atratus* (Waterhouse, 1875) and ab. gagates of *A.luridus* (Fabricius, 1775), especially to the former.

To facilitate the identification of *A.baei* sp. nov. and similar species discussed above; *A.atratus*, *A.humerospinosus*, *A.luridus*, and *A.superatratus* were photographed and their characters were compared in Table 1. The drawings of the body and aedeagus of *A.luridus* has been presented many times, e.g. by Balthasar (1963), Stebnicka (1976), and Bunalski (1999), but its epipharynx was first illustrated by Dellacasa et al. (2001), and the habitus photographed by Bunalski (1999) and Rössner (2012). The photographs of the habitus and aedeagus of *A.atratus* and *A.superatratus* were presented by Kawai et al. (2005). Here, we present, for the first time, photographs of the epipharynxes of all the species mentioned and the habitus and aedeagus of *A.humerospinosus*. We note that the shape of the aedeagi is a less distinctive character than the structure of epipharyngi, which differ considerably among species.

**Figures 47–56.**
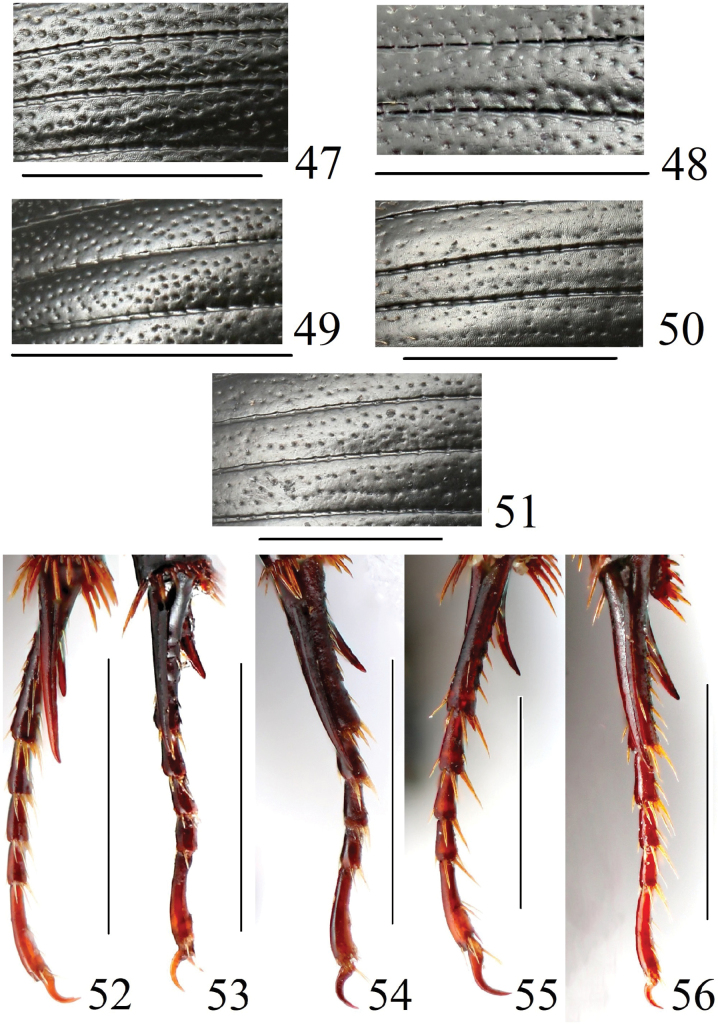
Elytra pattern and metatibiae of *Acrossus* species **47–51** elytra pattern **47***A.baei* sp. nov., ♂, holotype **48***A.atratus* (Waterhouse, 1875), ♂ **49***A.humerospinosus* (Petrovitz, 1958), ♂ **50***A.luridus* (Fabricius, 1775), ♂ **51***A.superatratus* (Nomura & Nakane, 1951). **52–56** metatibia **52***A.baei* sp. nov., ♂, holotype **53***A.atratus* (Waterhouse, 1875), ♂ **54***A.humerospinosus* (Petrovitz, 1958), ♂ **55***A.luridus* (Fabricius, 1775), ♂ **56***A.superatratus* (Nomura & Nakane, 1951), ♂. Scale bars: 1.0 mm.

**Figure 57.**
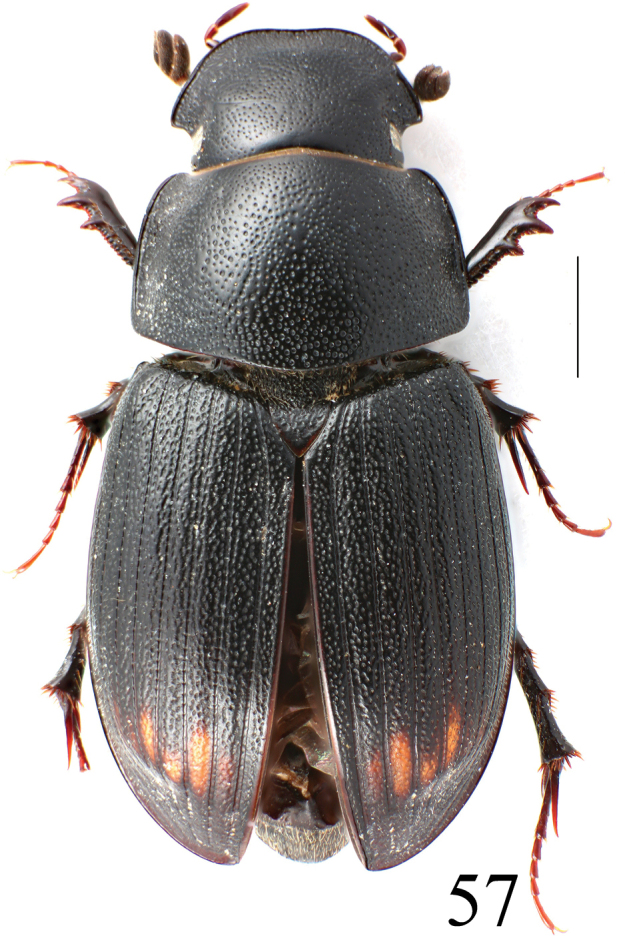
*Acrossusbaei* sp. nov., ♀, paratype, dorsal view. Scale bar: 1.0 mm.

## Supplementary Material

XML Treatment for Acrossus baei

XML Treatment for Acrossus atratus

XML Treatment for Acrossus humerospinosus

XML Treatment for Acrossus luridus

XML Treatment for Acrossus superatratus

## ﻿Additional information

### Author ORCIDs

Changseob Lim https://orcid.org/0000-0002-0565-5001

Łukasz Minkina https://orcid.org/0000-0001-7056-7334

### Data availability

All of the data that support the findings of this study are available in the main text.
